# Educational Needs of School Nurses Regarding the Evidence-Based Management of Sickle Cell Disease

**DOI:** 10.3390/ijerph182111641

**Published:** 2021-11-05

**Authors:** Lisa M Shook, Cami Mosley, Christina Bennett Farrell, Ann Connelly, Cheryl L Jones

**Affiliations:** 1 Cincinnati Comprehensive Sickle Cell Center, Division of Hematology, Cincinnati Children’s Hospital Medical Center, Cincinnati, OH 45229, USA; Cami.Mosley@cchmc.org (C.M.); Christina.Bennett@cchmc.org (C.B.F.); 2 Department of Pediatrics, College of Medicine, University of Cincinnati, Cincinnati, OH 45229, USA; 3 School Nursing and Early Childhood Health Programs, Ohio Department of Health, Columbus, OH 45229, USA; Ann.Connelly@odh.ohio.gov; 4 Sickle Cell Services Program, Ohio Department of Health, Columbus, OH 45229, USA; cheryl.jones@odh.ohio.gov

**Keywords:** sickle cell disease, school nurse, continuing education, evidence-based management, complications

## Abstract

Sickle cell disease (SCD) is a rare blood disorder that can have life-threatening complications. This presents a challenge for school nurses who may have had limited experience managing complications in the school setting. This study assessed the experience, self-reported knowledge, confidence and ability of school nurses in managing SCD in the school-setting and identified continuing educational needs and preferences. This study used a qualitative, descriptive approach. A survey was previously administered to over 400 school nurses who worked in K-12 schools in Ohio. Those participants who reported experience with managing SCD were invited to participate in a focus group or semi-structured interview. Data were interpreted using thematic analysis strategy. Four overarching themes emerged from the data: (1) perceived lack of support and resources, (2) self-reported lack of knowledge about SCD, (3) importance of partnerships with parents, and (4) need for continuing education and networking with other school nurses. Easily accessible, educational interventions, along with peer networking, can be designed to improve school nurse knowledge and confidence levels in managing SCD. These types of on-demand interventions are important as many school nurses reported infrequent exposure to students with SCD.

## 1. Introduction

School nurses play a vital role in providing comprehensive healthcare services to students during the school day, including those with chronic illnesses. School nurses are often faced with managing the complications students may experience in the school setting and should be prepared to treat those emergencies in the most effective way possible. However, researchers report that many school professionals, including school nurses, may not have adequate preparation, training, or knowledge to best help students with chronic illnesses [1].

Sickle cell disease (SCD), the most commonly inherited genetic disorder in the United States, affects the red blood cells and can affect individuals of African, Mediterranean, or Asian descent [2]. An estimated 100,000 individuals in the United States live with SCD and over 2000 newborns are diagnosed annually [3]. Children with SCD suffer from a wide variety of acute and chronic medical complications and may suffer serious morbidity, poor health-related quality of life, and early mortality. Moreover, children with SCD are at-risk for serious medical complications including acute chest syndrome, silent stroke, overwhelming sepsis rapidly leading to death, and pulmonary or cardiac complications [4]. Other potential SCD complications include sickle retinopathy, bone damage, priapism, and leg ulcers, as well as pain crises that require the administration of prescription pain medicine or even daily administration of hydroxyurea, a disease-modifying therapy [4]. Many of these complications are likely to arise during the school day, which requires school nurses to identify, knowledgeably and confidently, SCD emergencies and manage treatment appropriately [5]. However, there is a significant gap in the literature about school nurses and their knowledge, comfort, confidence and experience of managing SCD in the school setting.

The purpose of this study was to explore lived experiences of school nurses managing SCD in the school setting and to define the educational needs and preferences of school nurses.

## 2. Materials and Methods

### 2.1. Design and Setting

This qualitative study used focus groups and semi-structured interviews with a framework that was informed by the results of an Institutional Review Board approved school nurse survey conducted in spring 2019. The survey was distributed to 490 school nurses with an 83% survey completion rate (*n* = 407). The majority of the respondents were registered nurses (63%) with a bachelor’s degree (57%). Participants were pre-dominantly female (99%) and between the ages of 40–49 years old (32.5%). Most participants worked in public schools (86.8%), with over half working in more than one school location. In nearly 40% of the schools, students do not always have access to a school nurse during the school day. Only 35% of the respondents reported experience treating a student with SCD. Moreover, in regard to identifying SCD complications, 46% of respondents reported their ability to identify complications of SCD such as splenic sequestration, as “below average,” and only 14% of the school nurses had received continuing education about SCD in the past three years.

Focus group and semi-structured interview participants recruited were a convenience sampling who indicated on the School Nurse Survey (2019) that they had prior experience managing a student with SCD and were willing be contacted in the future. School nurses were recruited from Ohio schools (K-12). Participants were recruited by email and asked to complete an online scheduling survey. The focus groups and semi-structured interviews were held online utilizing the Zoom© web conference platform in January and February 2020. All participants were incentivized with $25 for their time.

### 2.2. Ethical Considerations

Permission to conduct the research (focus groups and semi-structured interviews) was obtained from the IRB at Cincinnati Children’s Hospital Medical Center and Logan University. Before the focus groups and semi-structured interviews began, participants gave oral informed consent, including voluntary participation and the right to withdraw at any time.

### 2.3. Data Collection

A trained focus group facilitator conducted the focus groups and semi-structured interviews with guides approved by the IRB. Focus groups were held online using Zoom © web-based video conferencing platform. Focus group discussions were led by the moderator using a script, lasted approximately an hour and was digitally recorded for professional transcription, while other trained staff took additional notes. Interviews were also conducted using Zoom© and digitally recorded. Recordings were professionally transcribed. The focus group script and interviews focused on the school nurses’ previous experience treating students with SCD as well as a discussion about the preferred methods and content of continuing education regarding SCD for school nurses.

### 2.4. Data Analysis

An inductive analysis based on a content analysis [6] was conducted. This included three phases: preparation, organizing, and reporting the data. In phase 1, focus group and interview transcripts were coded into main themes. During phase 2, data was further categorized into main categories and overall themes. An additional coder independently analyzed the transcripts and coding for quality assurance. A team meeting was held to debrief and discuss emerging findings. In the final analysis phase, coded text was reviewed for final themes.

## 3. Results

Two virtual focus groups were held with three participants each and semi-structured interviews were held with four participants (Table 1). 

Several main themes emerged, including lack of support for school nurses, lack of knowledge about SCD, the importance of partnerships with parents, and the need for continuing education and networking with other school nurses. Theoretical saturation was achieved. 

### 3.1. Lack of Support

Participants all agreed that school nurses face a number of challenges, including lacking support (i.e., funding, additional staff members) and busy workloads, as many of the nurses have high student ratios or rotate through many schools within a district.

“In my district, the biggest thing we also face, that I face, is lack of support, because I am the only nurse for the entire district. So, it’s very hard to fight for your students. You have to really fight for our students’ needs because they’re…we’re a minority in this school setting.”

Moreover, when school nurses are unavailable, unlicensed clinic aids with limited availability, the school secretary, or other staff members will step in to assist with minor clinic tasks. This was an important point, with school nurses noting that it was necessary to “reiterate with our staff…if that student (with SCD) comes in, please don’t give them ice (for pain). Let’s contact Mom. Please contact me.”

### 3.2. Lack of Knowledge, Confidence and Comfort Regarding SCD

Nurses reported limited knowledge and experience with students with SCD given the low number of students with the illness that they may encounter during their career, impacting their confidence and comfort level. Some nurses noted that their knowledge was limited to the pathophysiology of SCD that was briefly discussed in nursing school or previous nursing positions in hospitals. This often made it a challenge for nurses to discern if clinic encounters were true sickle cell emergencies.

“I personally have only had one student in 12 years of school nursing that’s had sickle cell. Prior to that, I worked in an emergency department. And so we handled it very differently than the emergency action plan that this mom came up with. So…I guess I was hesitant because I had always seen the other side of how it was handled versus what her protocol was. When we had a patient come into the emergency department, they went straight back to the room. We started grabbing labs. It was a whole protocol we followed versus whenever this student complained of any pain at all, or, of course, they had a fever- if they had a fever then that was different. But if they came in and had any pain at all, which down the road the student was in the clinic every day… Multiple times a week. And the biggest thing we wanted to remember was to not give ice and we would always call the mom right away. But it made it very, very hard to do our job, because it’s like ‘Well, she’s complaining of pain. Is it a crisis? Or is it just a normal ache and pain? Or does she just need a break?’

Another nurse confirmed that the infrequency with which they treat students with SCD contributes to a lack of confidence and comfort treating those patients.

“I would say my comfort level is very low … especially for an elementary aged child. I would say if it was a diabetic, I would put myself with 9/10 with being comfortable with him. But with sickle cell, I’d probably say 3 or 4. Just because I haven’t had a lot of experience with them.”

Lack of confidence was a concern; school nurses were not always confident about discerning when a younger student is having a complication with SCD.

“Because my kids are so young in my buildings, I mean, I want to trust my gut a lot, but then you always question yourself and don’t want to be wrong. I mean, if the kid comes down and says their legs hurt or they don’t feel good. And you’re like, ‘hmmm. I think they are tired. Wow. It’s amazing that they come at 1:15 every day and the teacher has shared that math is difficult’. But you always question yourself, because you’re not their parent. I mean, you don’t want to mess up or miss. Like my diabetics that I treat all the time, I know those kids well. I feel like I know them like their parents, but this is not something—we don’t see sickle cell crises. We don’t see our sickle cell kids every day. So therefore, it’s just not my area of comfort.”

There are also some common misconceptions about SCD among school nurses, including about which students may potentially have SCD.

“We have a very low percentage of African-American students in our district. That’s probably why we haven’t had very many kids with sickle cell.”

### 3.3. Need for Parental/Caregiver Partnerships

Several participants stressed the importance of having a working relationship with the student’s parent or caregiver in order to learn about the unique presentation of symptoms or complications with each student with SCD, since it can vary. Open dialogue with parents/caregivers about topics such as medication and treatment plans, complications, health history and emergency contact information can proactively inform school nurses’ decision-making and plan of action for managing health issues relating to SCD that may occur during the school day.

“I think that parents are a huge help…this parent is always available immediately to answer any questions and kind of triage it out… My student does not like to come to the nurse’s office. So, I often have to check on him and if Mom knows he’s not feeling well she will just call me up.”

One school nurse also reported a parent partnership monitoring a student’s progress on hydroxyurea, a disease-modifying therapy.

“… So I just know with this Mom we have been monitoring, like ‘Is he sick or are these symptoms from the medication?’ I’ve just been really good at charting and letting Mom know what the complaints are so she can relay that back to the child’s physician.”

Nurses agreed that collaborating with parents/caregivers to develop an IndividualHealthcare Plan, which is helpful for other chronic illnesses, such as diabetes, epilepsy and asthma, would be greatly beneficial for the care of students with SCD. Parent/caregiver involvement in writing the plan is essential.

“Anything they can share from the physician, and I feel the more you get from the parents too, if they would sit down and write notes. ‘This is what it looks like when my son has a crisis. This is what he usualy complains of. This is what I see at home. This (sic) complications we’ve had in the past. This is how many times he’s been hospitalized. This is what medications we’ve tried.’ Just as much information as possible.”

A participant raised an important point about inconsistency between provider treatment plans and parental/caregiver preferences for plans of action, which again points to the importance of a collaborative Individual Healthcare Plan that clearly outlines a course of action.

### 3.4. Need for Continuing Education

Participants all agreed that continuing education for evidence-based management of SCD is needed.

“I feel very strongly about being able to provide the best care I can for any student in our school district. I also want families to feel safe that the care provided for their student is by somebody who is knowledgeable and understands what sickle cell is. So I think it’s very important, the educational component, of learning what you can.”

Furthermore, participants agreed that resources that can be referenced on-demand are essential, as there may be a lapse of several years between participating in continuing education and encountering a student with SCD. A participant also suggested that engaging teachers in training about SCD is also necessary.

“Every year one of my biggest concerns is starting over. So every year they get a new teacher and you go through the education process again and the follow-up process because you have to ensure the teacher is educated on sickle cell. More times than not, they’ve never had a student with it. They don’t know anything about it, and you have to trust that you’ve trained that teacher because they’re in the classroom with them every day. They see all of those things first. They see all the signs before you do so you have to educate and trust that person to pay attention.”

Preferred continuing education modalities included conferences, online self-directed learning, speakers at schoolwide staff meetings, and online web resources that are easily accessible. Nurses explained that while comfort and confidence come from experience and knowledge, most school nurses typically do not have high frequency or a large volume of students with SCD, so combining education and networking with other nurses managing SCD virtually, such as using a tele-mentoring model that includes didactic educational presentations and de-identified case presentations by school nurses, may be greatly beneficial.

“I think that would be really interesting to see the different things that someone might encounter with a student with sickle cell. I think that would be pretty exciting, actually, to have that experience, and it’s not just ‘Oh this is what you look for.’ You know, to actually have the personal information about different cases. That would be exciting to see.”

## 4. Discussion

Knowledge and comfort of school nurses caring for students with SCD is highly dependent on past experiences. Because SCD is a rare disorder, many nurses have limited exposure to students and thus limited knowledge and comfort in treating them, while those with more frequent experience with SCD feel much more knowledgeable, confident and comfortable. This finding is consistent with that of other healthcare providers caring for SCD [7]. There is uncertainty among some school nurses about determining whether students are actually experiencing SCD complications or not, along with some hesitancy in identifying acute complications, such as pain or stroke. A few of the school nurses noted previous experience working as nurses in hospital settings and having negative interactions with patients with SCD, in some cases leading to an implicit bias towards patients with SCD, particularly around narcotics and pain management. This is an important dynamic to consider when developing educational interventions for school nurses. Studies have found that provider’s attitudes and biases toward sickle cell patients in pain can be a barrier to optimal pain management [8]. 

Individual Healthcare Plans (IHP) are essential to providing consistent and evidence-based care to students with chronic illnesses. Engelke et al. [9] have shown that working with families to develop an IHP for students with chronic illnesses, including SCD, led to improved quality of life, grades, participation in classroom and extracurricular activities, and overall disease management. Moreover, many school nurses rotate to several schools, leaving other school administrative staff to step in to provide clinic coverage. Having an IHP in place is helpful for other staff members, as well as teachers and substitute teachers, to provide context to the student’s health condition and individual treatment plan, including pain management. Ideally, these IHPs are written collaboratively by the student’s parent/caregiver, healthcare team, and school nurse. Parental/caregiver involvement is important for building a trusting relationship to provide high-quality care for students with SCD. A template of key talking points for working with parents/caregivers of students with SCD may also be a helpful tool for school nurses.

School nurses all reported a need for continuing education on SCD and suggested tools that develop the ability to identify complications and evaluate pain. Nurses expressed a desire for online, self-paced modules, as online education not only removes many of the barriers of attending conferences, but also can be accessed on-demand when school nurses encounter students with SCD. School nurses report searching online for evidence-based best practices, often on local children’s hospital websites. 

Innovative educational approaches that leverage technology, such as Project ECHO© tele-mentoring, may successfully provide education and build a community of practice among school nurses about SCD. Project ECHO© has been successfully implemented to educate other interdisciplinary healthcare providers regarding SCD [10]. School nurses all echoed the need for continued education, but also emphasized the importance of networking with others. The Project ECHO© format is designed to be a one-hour virtual continuing education event that includes a 15–20-min educational didactic presentation, as well as time for one or two de-identified case presentations that would allow school nurses to elicit recommendations for other participants about how to manage a specific situation with a student with SCD. Tele-mentoring reduces the barriers of cost, time, travel, or the need for arranging coverage for school nurses attending a conference, since it is virtual.

## 5. Conclusions

While SCD is the most common genetic disorder in the United States, many school nurses may not have previous exposure to this population and the complex health challenges that may occur. This presents a unique challenge to developing timely continuing education interventions that can be easily accessed by school nurses when needed. Although a potential limitation of this study is the small number of participants, many important themes emerged and are likely to reflect universal needs of school nurses. However, the gaps in the literature present the opportunity to continue to expand on exploring the educational needs and experiences of school nurses with SCD. This study has created an initial framework to understand the gaps in continuing education for school nurses and point to some potential education interventions. Outcomes of this study imply that the implementation of interventions, such as Project ECHO © tele-mentoring, may be useful to remotely provide education and create a community of practice among school nurses as well as other school staff, when managing students with SCD. Beyond tele-mentoring, on-demand learning, such as self-directed learning modules, and resources such as templates for IHPs for SCD are highly preferred to meet the immediate learning and practical needs of school nurses in real-time when encountering students with SCD.

## Figures and Tables

**Table 1:** Demographic characteristics of school nurse focus groups and interview participants.

	Age	Education Level	Years of Exp.	Home School	Public/Private
Nurse #1	40–49	Associate Degree, RN	10	Pre-K, Elementary	Public
Nurse #2	30–39	Master’s Degree, RN	8	Elementary	Public
Nurse #3	40–49	Bachelor’s Degree	1.5	Pre-K, Elementary, Middle	Private
Nurse #4	50–59	Master’s Degree	27	Elementary, Middle School	Public
Nurse #5	50–59	Bachelor’s Degree	7	Elementary, High School	Public
Nurse #6	30–39	Bachelor’s Degree	1	Elementary, High School	Public
Nurse #7	40–49	Bachelor’s Degree, RN	21	Pre-K—High School	Public
Nurse #8	40–49	Master’s Degree. RN	3.5	High School	Public
Nurse #9	30–39	RN	7	Elementary School	Public
Nurse #10	60+	Associate Degree, RN	8	Elementary School	Public

## Data Availability

The data presented in this study are available on request from the corresponding author.
